# Comparative analysis of cystic biliary atresia and choledochal cysts

**DOI:** 10.3389/fped.2022.947876

**Published:** 2022-08-24

**Authors:** Yu-tong Chen, Ming-juan Gao, Ze-bing Zheng, Lu Huang, Qing Du, Dai-wei Zhu, Yuan-mei Liu, Zhu Jin

**Affiliations:** ^1^Department of Pediatric Surgery, Affiliated Hospital of Zunyi Medical University, Zunyi, China; ^2^Department of Pediatric Surgery, Guizhou Children’s Hospital, Zunyi, China

**Keywords:** cystic biliary atresia, choledochal cyst, direct bilirubin, cyst, early surgical treatment type of study

## Abstract

**Objective:**

Cystic biliary atresia (CBA) is a rare and peculiar type of biliary atresia (BA) that is easily confused with infantile choledochal cysts (CCs). This study explored information for early CBA diagnosis and treatment.

**Method:**

The authors retrospectively analyzed the clinical data of 32 children with hilar cysts from January 2013 to May 2021. According to the diagnosis, they were divided into the CBA (*n* = 12) and CC (*n* = 20) groups. Patient features, biochemical indexes, preoperative ultrasound characteristics, cholangiography features, and intraoperative findings were analyzed and compared between the two groups.

**Results:**

The alanine aminotransferase, aspartate aminotransferase, total bilirubin, and direct bilirubin levels in the CBA group were higher than in the CCs group (*P* < 0.05). Additionally, B-mode ultrasound showed a cystic mass in front of the hepatic hilum, and the cyst size was much smaller in the CBA group compared with the CC group (2.2 ± 1.3 cm vs. 6.0 ± 2.2 cm, *P* < 0.001). Among all of the parameters, cyst width was the most accurate for identifying CBA and CCs. A cutoff value of 2.5 cm (area under the curve, 0.98, *P* < 0.001) showed 90.9% sensitivity and 95% specificity for cyst size.

**Conclusion:**

For children with early-onset severe jaundice, and if the width of the cystic mass was ≤2.5 cm, a diagnosis of CBA was highly likely. Early cholangiography and surgical treatment are necessary for the effective treatment of these infants.

## Introduction

Biliary atresia (BA) is a disease characterized by severe jaundice caused by biliary obstruction in infancy. The disease etiology is unclear (1) and its incidence varies in different regions, i.e., approximately 1/18,000 in Europe and higher in Asia, where it is approximately 1/5,000 (2). Biliary atresia can be divided into three types (3). Cystic BA (CBA) is a rare and unusual type of this disease (4, 5) that manifests as cystic changes in the hepatic hilum of patients with BA, accounting for approximately 5–10% of the total number of BA cases (6). The application of prenatal ultrasound screening in the differential diagnosis of CBA and choledochal cysts (CCs) can increase the detection rate of cystic hilar masses (7). Based on the significant similarity between CCs and CBA, the two diseases can easily be confused, leading to misdiagnosis. However, timely surgical intervention and selecting the correct procedure can significantly influence patient prognosis (8). This study aimed to explore diagnosing and treating CBA compared with CCs to ultimately improve the patient prognosis of the former. Early surgical treatment (less than 60 days) can improve the survival native liver rate in CBA cases and assist pediatric surgeons in differentiating between the two disorders.

## Materials and methods

This retrospective study included 12 cases of CBA and 20 CCs cases from the Affiliated Hospital of Zunyi Medical University from January 2013 to May 2021. A total of 12 patients with CBA were admitted for jaundice and clay-colored stool. Among the 20 CBD children, 4 cases were admitted due to yellow skin staining, 3 cases due to light stool color, and 13 cases due to the presence of a hilar cyst, discovered by prenatal ultrasound or physical examination. All patients were finally diagnosed by intraoperative cholangiography (IOC) and pathological examination. Each patient’s age, sex, and routine biochemical examination results (including the levels of total bilirubin [TBIL], direct bilirubin [DBIL], alanine aminotransferase [ALT], aspartate aminotransferase [AST], and gamma-glutamyltransferase [GGT]) were recorded preoperatively. The following data were obtained from B-mode ultrasound, magnetic resonance imaging (MRI), and intraoperative examinations: the size and shape of the gallbladder, the size of the liver hilar cyst, and the presence/absence of the contrast agent in intra- and extrahepatic biliary tracts and cysts. Color ultrasound examination of the gallbladder and cyst sizes and examination methods were as follows: the length of the gallbladder was taken as the largest length of the lumen, the width of the gallbladder was the largest width of the lumen, and the size of the cyst was described by the largest length and width of the cyst. All children fasted for more than 4 h before the ultrasound examination. An MRI examination was performed for all patients. Two experienced pathologists, who were unaware of the collected data for clinical analysis, independently performed a retrospective examination of the liver specimens.

The operative indications of CBA were persistent jaundice, clay-colored stools, increased TBIL, and DBIL/TBIL 50%. The operative indication for CCs was the presence of a cystic mass at the hilar area of the liver, which is typically treated after the first month. The same surgeon performed all of the surgeries in this study.

IOC showed that the intrahepatic and extrahepatic bile ducts were not development, and a cystic mass was found while exploring the hilum of the liver. The proximal end of the mass formed fibrous plaque in the hilar atresia, which contained colorless or yellowish fluid. Postoperative liver pathology showed bile duct hyperplasia and cholestasis, which was subsequently diagnosed as CBA. Furthermore, IOC showed that the intrahepatic and extrahepatic bile ducts were development, and postoperative pathology showed no or slight bile duct hyperplasia, which was subsequently diagnosed as CCs.

The SPSS Statistics 23.0 software program was used for data analysis in this study. Data that were subject to normal distribution were expressed as mean ± standard deviation (SD), and a Student’s t-test was adopted according to the specific context. Non-normal distribution was represented by the median (range). Non-parametric tests (e.g., a Wilcoxon rank-sum test) were also conducted, and *P* < 0.05 was considered statistically significant. Additionally, receiver operating characteristic (ROC) curve analysis was applied to analyze the indicators that showed significant differences.

## Results

### Patient characteristics

A total of 32 children (12 with CBA and 20 with CCs) were included in this research. There were 5 males and 7 females in the CBA group and 2 males and 18 females in the CC group. The mean age at the time of surgery was 65.58 ± 27.10 days (84.75 ± 37.72 days in the CBA and CC groups, respectively). All children were from Guizhou Province, and there was no statistical significance in sex, weight, or age between the two groups (Table 1). One child in the CBA group had congenital heart disease.

**TABLE 1:** Comparison of general gallbladder conditions between the CBA and CC groups.

Parameters	CBA (*n* = 12)	CC (*n* = 20)	*P-* value
Age (days)	65.6 ± 27.1	84.8 ± 37.7	0.135
Sex (female/male)	7/5	2/18	0.073
Weight (kg)	5.1 ± 1.4	5.5 ± 1.6	0.394
Jaundice	9/12 (75%)	6/20 (30%)	0.002
Clay stool	9/12 (75%)	3/20 (15%)	0.027

### Laboratory examinations

Biochemical tests were carried out for the patients with CBA and CCs. The laboratory examinations indicated that the TBIL level was significantly increased in the CBA group, mainly due to DBIL. The authors also measured the ALT, AST, and GGT levels in both groups. In the CBA and CC groups, respectively, the median TBIL was 170.9 and 43.9 μmol/L, the median DBIL was 99.9 and 23.9 μmol/L, the median ALT was 117 and 47 U/L, the median AST was 210.5 and 90.5 U/L, and the median GGT was 440.5 and 275.5 μmol/L. Concerning the biochemical outcomes, except for GGT, the levels of ALT, AST, TBIL, and DBIL in the CBA group were all higher compared with the CCs group (*P* = 0.91), and there was a significant difference between the two groups (*P* < 0.001). There was no significant difference in the white blood cell count, percentage of neutrophils and lymphocytes, red blood cell count, or hemoglobin levels between the two groups (Table 2). The ROC curve analysis showed that the area under the curve (AUC) for TBIL, in terms of differentiating CBA from CCs, was 0.88 with sensitivity and specificity of 91.67 and 85%, respectively, when TBIL was ≥98 μmol/L (Table 3).

**TABLE 2:** Comparison of clinical and biochemical information between the CBA and CC groups.

Characteristics	CBA (*n* = 12)	CC (*n* = 20)	*P-* value
TBIL (μmol/L)	170.9	43.9	< 0.0001
DBIL (μmol/L)	99.9	23.9	< 0.0001
ALT (U/L)	117.0	47.0	0.003
AST (U/L)	210.5	90.5	< 0.0001
GGT (U/L)	440.5	257.5	0.144
WBC	9.8	9.6	0.969
NEUT%	0.2	0.2	0.349
NEUT (10^9^/L)	2.4	2.2	0.508
LYN%	0.6	0.6	0.520
LYN (10^9^/L)	5.4	6.4	0.436
RBC (10^12^/L)	3.6	3.5	0.938
HBG (g/L)	108.5	102.5	0.311
Length of gallbladder	2.7 ± 1.2	5.0 ± 1.8	< 0.0001
Width of gallbladder	0.6 ± 0.2	1.8 ± 1.1	< 0.0001
Length of cyst	2.2 ± 1.3	6.0 ± 2.2	< 0.0001
Width of cyst	1.6 ± 0.8	5.1 ± 1.9	< 0.0001
Length of cyst on color ultrasound	2.4 ± 1.1	6.4 ± 3.5	< 0.0001
Width of cyst on color ultrasound	1.5 ± 1.0	4.4 ± 2.0	< 0.0001

ALT, alanine aminotransferase; AST, aspartate amino-transferase; CBA, cystic biliary atresia; CC, choledochocyst; DBIL, direct bilirubin; GGT, gamma-glutamyltransferase; DBIL, indirect bilirubin; TBIL, total bilirubin; WBC, white blood cell; NEUT, neutrophil; LYN, lymphocyte; HBG, hemoglobin; RBC, red blood cell.

**TABLE 3:** Differences in the diagnostic performance of parameters.

Characteristics	AUC (n)	Sensitivity (%)	Specificity (%)	Cutoff (n)	*P-* value
TBIL	0.88	91.7	85.0	98.0	< 0.0001
DBIL	0.86	83.3	80.0	58.9	< 0.0001
ALT	0.71	75.0	65.0	74.0	0.0302
AST	0.81	75.0	85.0	171.0	0.0001
Length of gallbladder	0.87	81.8	79.0	3.0	< 0.0001
Width of gallbladder	0.94	90.9	89.5	0.8	< 0.0001
Length of cyst	0.94	90.9	85.0	3.2	< 0.0001
Width of cyst	0.98	90.9	95.0	2.5	< 0.0001
Length of cyst on color ultrasound	0.88	90.0	78.6	3.2	< 0.0001
Width of cyst on color ultrasound	0.91	80.0	85.7	2.4	< 0.0001

ALT, alanine aminotransferase; AST, aspartate aminotransferase; AUC, area under the curve; DBIL, direct bilirubin; TBIL, total bilirubin.

### B-mode ultrasound and magnetic resonance imaging

**FIGURE 1:**
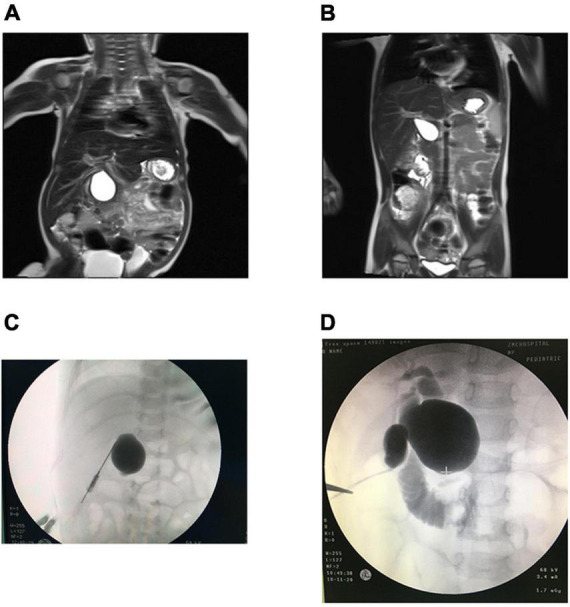
MRI in CBA and CC patients. **(A)** CBA: hepatic hilar cyst. **(B)** CC: well-circumscribed, preportal cyst. IOC in CBA and CC patients. **(C)** CBA: only cysts were visualized. **(D)** CC: the gallbladder, cyst and duodenum were visualized.

B-mode ultrasound was performed for both groups before surgery. The authors used this method to observe and record the gallbladder shape, change in gallbladder size after a meal compared with before, and discern the cyst in front of the hilum. In the CBA group, the gallbladder was not detected in 8 cases, and exploration of the cystic duct at the gallbladder fossa was performed in 4 cases. No significant change in the gallbladder was observed after compared with before a meal. In 20 cases in the CC group, the gallbladder size was within the normal range; for most patients, the gallbladder length was 4–6 cm and the width was 1.5–2.5 cm. There was a significant difference in the width and length of cysts between the CBA and CC groups (1.5 ± 1.0 vs. 4.4 ± 2.0 cm and 2.4 ± 1.1 cm vs. 6.4 ± 3.5 cm, respectively, *P* < 0.001, Table 1). The ROC curve analysis showed that the AUC for ultrasound identification of the cyst length in the CBA and CC groups was 0.88 at an optimal cutoff of 3.2 cm, with sensitivity and specificity of 90% and 78.6%, respectively. A 2.5 cm cutoff value for cyst width (AUC, 0.98, *P* < 0.001) showed sensitivity of 90.9% and specificity of 95% for cyst size (Table 3). Magnetic resonance imaging clearly showed a round, well-margined cystic mass in front of the hepatic hilum in the CBA (Figure 1A) and CC (Figure 1B) groups.

### Intraoperative cholangiography and findings

Cholangiography was performed through the gallbladder during surgery in the CBA group and showed no intra- or extrahepatic biliary imaging findings (*n* = 7); in some cases, cyst imaging findings were not accompanied by biliary imaging findings (*n* = 5) (Figure 1C). If the gallbladder, cyst, intra- and extrahepatic bile ducts, and intestines could be visualized, CC was diagnosed, otherwise, CBA was confirmed. Surgical exploration suggested that the cysts in the CBA group were smaller (*n* = 5), had necrotic tissue (*n* = 1), and included a small amount of bile-like material (*n* = 6). However, contrast media were found to have passed through the biliary tract in the CC group (Figure 1D).

**FIGURE 2:**
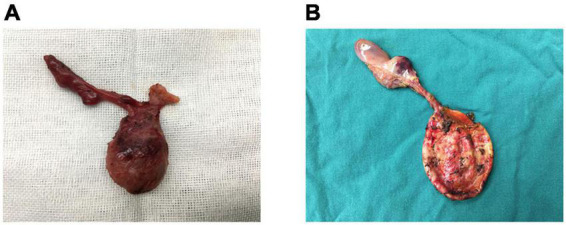
**(A)** CBA: gallbladder dysplasia, a fibrous mass, and the largest cyst of all CBA cases (3 cm). **(B)** CC: a normal gallbladder, no hilar fibrous mass, and a large cyst.

The 12 patients with CBA were treated using the Kasai procedure, and cysts were located by hepatic portal probing, referencing the findings on color ultrasound. After cutting the cyst, its wall was observed to have been observably thickened with edema, and a small amount of bile pigment was visible. A small amount of a yellow, bile-like substance was found in the cyst in 8 cases, stereo cysts were found in 3 cases, and necrotic tissue without bacteria was found in 1 case, with no intrahepatic biliary connections. The width of the cyst was much smaller in the CBA than in the CC group (1.6 ± 0.8 vs. 5.1 ± 1.9 cm, *P* < 0.001, Table 3). One of the 12 patients with CBA had a very large cyst, approximately 3.5 cm in length, with a narrow and small gallbladder and characteristic fibrous plaques (Figure 2A). The patients with CC had relatively large cysts, a regular gallbladder size, and a smooth gallbladder wall with normal wall thickness (Figure 2B).

### Patient outcomes

All 20 children with CCs recovered completely and their liver function returned to normal. Among the 12 children with CBA, 6 cases had normal levels of bilirubin 3 months after surgery, 4 cases underwent liver transplantation 6 months after surgery, 1 case died 3 months after surgery, and 1 case was lost to follow up.

## Discussion

BA and CCs are common causes of obstructive jaundice. CBA is a particular type of BA with a generally good prognosis (9). A total of 320 patients with CCs had been treated at the authors’ hospital during the past 8 years, but only 20 patients who received surgical treatment were less than 150 days old. Nearly all children with CBA underwent surgical treatment within less than 130 days following birth. This was primarily because without early surgical intervention, children with BA rapidly develop cirrhosis, which can be life-threatening, while children with CCs exhibit some or no discomfort until they are diagnosed through the discovery of abdominal masses (10). CBA can easily be misdiagnosed as CCs prior to surgery (11), even though ultrasound can identify cysts. There are also significant differences between the surgical methods that are applied to the two different conditions; the Kasai procedure is performed for CBA, while cyst resection and choledochojejunostomy are performed for CCs. This misunderstanding leads to untimely surgical intervention, which can affect the prognosis and even threaten the lives of children. Accordingly, early detection, diagnosis, and treatment are critical.

In this study, 12 patients with CBA presented with significant jaundice, hepatic impairment, and an elevated TBIL level, particularly in terms of DBIL (12–15) in infancy (16). During the same period, the lesions affecting liver function in children with CCs were milder than those in children with CBA, which was likely the cause of the less critical cholestasis. The ALT, AST, TBIL, and DBIL levels in the CBA group were all significantly higher than those in the CC group in this study, which was consistent with the results reported in several existing studies (17). This indicated that these indexes could be used as sensitive indicators to distinguish CBA from CCs. However, it has also been reported that GGT cannot be used as a clinical indicator to differentiate CBA from CCs (18). Chen et al. (19) proposed that the GGT level could effectively be used to diagnose BA in children younger than 120 days. However, due to the limited sample size of the current study, some confounding factors were not excluded; as such, additional data are required for further verification.

Color Doppler ultrasound is a non-invasive, rapid, highly sensitive, and specific method for detecting obstructive jaundice (20). Kanegawa et al. (21) reported that when the gallbladder was less than 15 mm in size, this condition could be used as a standard for diagnosing BA. The current authors’ observations suggested that in cases of CBA, the gallbladder may be atresic, dysplastic, or cord-like, whereas the gallbladder in patients with CC will show normal development, a result that was consistent with relevant reports (22) (Figure 2A). The current authors found that the size of the hilar cysts in patients with CBA was significantly smaller than in patients with CC, with the critical value for identification being a length of 3.2 cm (Figure 2B). This result may have been due to lower bile secretion or biliary obstruction in infancy in CBA cases.

Existing reports suggested that CBA should be considered when the cyst size is <1.5 cm (23). The parameter with the highest accuracy and significant diagnostic performance for distinguishing CBA from CCs is cyst size. Meanwhile, according to the data analysis, if the length of the gallbladder is <3 cm or its width <0.8 cm (as indicated by ultrasound), a CBA diagnosis will be highly likely. For larger CBA, as well as the ultrasonic measurement of the maximum width and length of the gallbladder and its length-to-width ratio, the maximum length and width, as well as the aspect ratio of the cyst may be useful for a diagnosis of CBA.

Incising the cyst wall will reveal a large amount of dark-green bile and biliary sludge in patients with CC and only a small amount of yellow fluid in patients with CBA (24). The CCs typically present as large cysts with a large amount of bile and biliary sludge; the gallbladder is typically not atresic, the hepatic duct is more likely to be dilated, and contrast media will be able to enter the duodenum, which cannot be found in patients with CBA (Figure 1D).

Intraoperatively, 1 patient with CBA exhibited aseptic necrotic tissue in the cyst due to the cyst’s incompatibility with the intra- and extrahepatic bile ducts, resulting in cholestasis and the gradual formation of biliary sludge. Minor cysts, indicators of fibrous masses, gallbladder atrophy, and irregular cholecystic walls are characteristic features of CBA on ultrasound, and these were similarly observed during the surgical exploration of the current authors.

Whether there are specific and transparent criteria for “early surgery” in cases of CBA remains controversial. For example, some scholars believe that earlier surgery is always better. Studies have shown that early interventional Kasai procedures (<70 days of age) (25) could improve biliary drainage and result in better efficacy in patients with CBA than in isolated BA (26). However, reports indicated that the therapeutic effect of surgery was lower when performed within 60 days of age compared with an age older than this (27). Although there is no uniform understanding of the optimal age for surgery, the current authors believe that early surgery (60–90 days of age) is vital for restoring bile flow, controlling the development of persistent inflammation, and slowing the progression of cirrhosis. Based on this perspective, a good prognosis depends on early surgical treatment.

Due to the limitations of ultrasound and biochemical examinations, these methods cannot be used as a gold standard for the definitive diagnosis of CBA or CCs. In this context, laparoscopic exploration is a means of investigation alongside surgical treatment. Laparoscopic examination and intraoperative biliary tract imaging can thus be performed early on to make a definitive diagnosis of either CBA or CCs. Long-term follow up has shown that only 30–50% of patients experience an excellent permanent prognosis (28). Postoperative complications, such as recurrent cholangitis and cirrhosis, remain prevalent in most children, which may be related to long-term inflammation and immune responses (10) leading to liver damage before surgery. Liver transplantation remains the only treatment for liver failure in the late stage of BA.

This study includes some limitations due to its small number of cases and because all the patients were selected from a single center in Zunyi, Guizhou. The study’s regional distribution of diseases varied significantly, and Asian individuals were more likely to be affected. Therefore, the results of this study may only apply to children in this particular region. Data from multiple centers are needed to confirm the findings of this study in future research. Only 12 children received a final diagnosis of CBA by IOC at the authors’ hospital during the past eight years, which may have been related to a reduced incidence, and could have affected the results of the ROC curve analysis. Therefore, the conclusions drawn in this paper require further confirmation through studies with larger samples.

## Conclusion

In summary, CBA should be considered in children with early-onset severe jaundice, caused primarily by DBIL elevation and cystic masses that are smaller than 2.5 cm in width and situated at the hepatic hilum. CBA requires early cholangiography and surgical treatment.

## Data availability statement

The original contributions presented in this study are included in the article/supplementary material, further inquiries can be directed to the corresponding author.

## Ethics statement

The studies involving human participants were reviewed and approved by Ethics Committee of Affiliated Hospital of Zunyi Medical University. The patients/participants provided their written informed consent to participate in this study.

## Author contributions

Y-TC and ZJ: conception and design of the research. Y-TC and M-JG: acquisition of data. Z-BZ and ZJ: analysis and interpretation of the data. LH, QD, and ZJ: statistical analysis. ZJ: obtaining financing. Y-TC and D-WZ: writing of the manuscript. Y-ML and ZJ: critical revision of the manuscript for intellectual content. All authors read and approved the final draft.
